# A Simple Model to Estimate the Percentage of Motor Plan Reuse From Hysteresis Effect Size

**DOI:** 10.3389/fpsyg.2019.00561

**Published:** 2019-03-14

**Authors:** Christoph Schütz, Thomas Schack

**Affiliations:** ^1^Cluster of Excellence Cognitive Interaction Technology, Bielefeld University, Bielefeld, Germany; ^2^Faculty of Psychology and Sports Science, Bielefeld University, Bielefeld, Germany; ^3^CoR-Lab, Research Institute for Cognition and Robotics, Bielefeld University, Bielefeld, Germany

**Keywords:** motor planning, motor hysteresis, motor plan reuse, modeling, sequential task

## Abstract

In sequential tasks, a partial reuse of former motor plans results in a persistence in the former posture (termed hysteresis). The *cost-optimization* hypothesis states that the percentage of reuse depends on the relative cognitive and mechanical cost of each movement. These costs should be constant across all drawers, yet previous studies found a larger hysteresis effect at the central drawers and declining effects toward the periphery. In the current study, we show that a simple mathematical model that assumes a sigmoid optimal grasp angle function and a fixed percentage of motor plan reuse explains the posture variance in a randomized and an ordered sequential drawer opening task. This finding indicates that (1) the optimal pro/supination angle is a sigmoid function of drawer height, (2) the percentage of motor plan reuse is constant across drawers, and (3) a constant percentage of reuse results in a larger hysteresis effect at the central drawers. Based on the model, the percentage of motor plan reuse in future studies can be estimated from the size of the motor hysteresis effect.

## Introduction

When reaching for a cup of coffee, we are unaware of the series of sensorimotor transformations that are necessary to translate the intended perceptual effect of grasping the cup into a muscle activation pattern that guides our hand to its final position. Due to these transformations, the creation of each motor plan is associated with a cognitive cost. In a repetitive motor task, we therefore do not create a new motor plan for each movement, but instead reuse (and modify) the previous plan (Rosenbaum and Jorgensen, 1992). Such a reuse manifests in a persistence in the previous posture.

For example, if participants have to open a column of drawers in a descending sequence, they adopt a pronated initial posture for the highest drawer and persist in a more pronated posture throughout the sequence. In an ascending sequence, they start in a supinated posture at the lowest drawer and subsequently persist in a more supinated posture (Schütz et al., 2011). The posture adopted at each drawer thus depends on the movement direction, which indicates a partial reuse of the previous motor plan (termed *motor hysteresis*; cf. Kelso et al., 1994). Motor hysteresis in posture selection has been reliably reproduced in a large number of studies (Rosenbaum and Jorgensen, 1992; Weigelt et al., 2009; Schütz et al., 2011, 2016; Schütz and Schack, 2013).

Motor hysteresis can be formally explained by dynamic systems theory (Haken et al., 1985). Recent studies, however, support the idea that hysteresis reflects a cognitive aspect of motor planning (van der Wel et al., 2007; Weigelt et al., 2009). Van der Wel et al. (2007) had participants contact a series of targets on a tabletop in time with a metronome. An obstacle had to be cleared between two targets. Jump peak height after the obstacle decreased only gradually. This hysteresis effect even persisted if participants cleared the obstacle with one hand and continued the progression with the other, thus discarding dynamic systems theory as the sole explanation for motor hysteresis.

**FIGURE 1:**
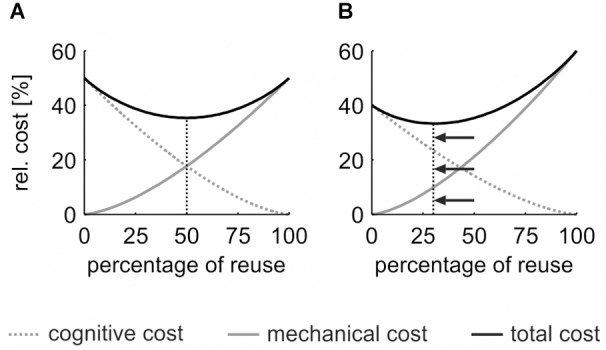
Model of the *cost-optimization* function. **(A)** Cognitive cost (*dotted gray line*) decreases with the percentage of reuse, mechanical cost increases (*solid gray line*). Cost factors follow a power function x^p^ (|*p*| > 1). The sum of both cost factors (*solid black line*) is minimized at an optimal percentage of reuse. **(B)** An increase of the mechanical cost (relative to the cognitive cost, e.g., 60:40) shifts the optimal percentage of reuse to a lower value that ensures minimal overall movement cost.

Instead, the cognitive cost of movement planning seems to be the cause of the hysteresis effect. To reduce the planning cost, previous motor plans are partially reused (Rosenbaum et al., 2007). A larger percentage of plan reuse reduces the cognitive cost of motor planning (Figure 1A, *dotted gray line*) but results in a larger persistence in the previous posture (Schütz et al., 2016), which in turn increases the mechanical cost of movement execution (Figure 1A, *solid gray line*). The *cost-optimization model* (Schütz et al., 2016) assumes that the sum of both cost functions (Figure 1A, *solid black line*) is minimized at one specific point, where the descent of the cognitive cost function equals the ascent of the mechanical cost function. This “equilibrium point” reflects the optimal percentage of reuse for the task and, thus, determines the size of the motor hysteresis effect.

The *cost-optimization model* predicts that changes in the percentage of reuse result from a change in the relative cognitive or mechanical cost. For example, if the mechanical cost of a task increases relative to the cognitive cost (e.g., 60:40, see Figure 1B), the “equilibrium point” that yields the minimal overall movement cost shifts to a lower percentage of reuse (cf. shift from Figure 1A to Figure 1B). This in turn reduces the size of the hysteresis effect, which serves as a proxy for the percentage of reuse. The model prediction was verified in a previous study (Schütz and Schack, 2013): participants executed a sequential drawer opening task. A hysteresis effect was found in the baseline condition. After increasing the mechanical cost of the task for 10 sequences, the size of the hysteresis effect was significantly reduced.

A potential shortcoming of the *cost-optimization model* is that, in a sequential drawer opening task, no changes in mechanical or cognitive movement cost should occur, as the complexity of the motor plan and the mechanical work required to open and close each drawer are comparable across drawers. Yet, drawer studies repeatedly reported a significant interaction of “sequence” (a/descending) × “drawer” height (Weigelt et al., 2009; Schütz et al., 2011, 2016; Schütz and Schack, 2013), resulting from a large hysteresis effect at the central drawers and declining effects toward the periphery. As no differences in cognitive and mechanical cost were present between central and peripheral drawers, should the hysteresis effect not be similar if the *cost-optimization model* held true?

Interestingly, all mentioned drawer studies also found a sigmoid relationship between drawer (height) and posture (Weigelt et al., 2009; Schütz et al., 2011, 2016; Schütz and Schack, 2013), with a steep slope at the central drawers and shallower slopes toward the periphery. Thus, the difference in posture between two subsequent drawers is highest at the central drawers. If we assume that (1) the optimal pro/supination angle (minimal mechanical cost of movement execution) for each drawer is a sigmoid function of drawer height and (2) the percentage of reuse is constant across drawers, a constant percentage of reuse would (3) result in a higher hysteresis effect at the central drawers.

To test these assumptions, we asked participants to execute a drawer opening task. Pro/supination of the hand was measured as the dependent variable. In randomized sequences of drawers, postures are unaffected by the previous posture (Schütz et al., 2011). Therefore, participants should create a motor plan from scratch for each drawer and adopt the (mechanically) optimal posture. We hypothesize that a sigmoid model (1) should capture a major fraction of the data variance in a randomized task.

In ordered (a/descending) sequences of drawers, each motor plan is created from the previous motor plan. We hypothesize that a sigmoid model (1) with a constant percentage of motor plan reuse (2) between subsequent drawers should capture a major fraction of the variance of “drawer” (sigmoid optimal posture), of “sequence” (hysteresis effect), and of the interaction of “sequence” × “drawer” (higher hysteresis effect at the central drawers, 3).

If the percentage of reuse was constant across drawers, the size of the hysteresis effect should increase if the spatial difference and, thus, the difference in optimal posture, between subsequent drawers increased. To test this, we asked participants to execute an ordered (a/descending) drawer opening task, but to skip every second drawer. If the percentage of reuse depended only on the cognitive and mechanical cost of the task, we expected a larger hysteresis effect in the skipped task compared to the normal task (4). This should be reflected by an interaction of “task” × “sequence.”

If the model is adequate for randomized, ordered, and skipped tasks, one can reasonably assume that it can be used to measure the percentages of motor plan reuse and novel planning in any sequential task. For example, it could be applied (1) to test how proposed hemispheric differences in cognitive (Janssen et al., 2009, 2011) or mechanical costs (Sainburg and Kalakanis, 2000; Sainburg, 2002) of a movement affect the optimal percentages of reuse in the dominant and non-dominant limb, or (2) to test if changes in working memory (WM) load interfere with motor planning: WM models postulate distinct verbal and spatial memory stores (Baddeley and Hitch, 1974; Baddeley, 1992) and attribute motor planning to spatial WM (Logie, 1995; Lawrence et al., 2001). Based on the model one could test if a depletion of spatial WM resources by a concurrent spatial memory task reduces novel planning in a sequential motor task.

The proposed modeling approach is imperative for sequential posture selection tasks. The percentage of reuse in hand path planning tasks (Jax and Rosenbaum, 2007, 2009; van der Wel et al., 2007) can be easily calculated: if, for example, participants have to clear an obstacle while contacting targets in a serial progression, jump peak height gradually returns to a baseline (van der Wel et al., 2007). From the gradient of this exponential decay, the percentage of reuse can be calculated. In posture selection tasks, on the other hand, no common baseline exists. To calculate the percentage of reuse, the last posture has to be fused with an optimal posture that varies between trials, which can be done by our model. The required optimal posture for any task and all target positions can be determined by a series of randomized sequences, which are unaffected by hysteresis (Schütz et al., 2011).

## Materials and Methods

### Participants

Thirty-one students [19 female, 12 male, age 24.5 ± 3.3 (SD) years] from Bielefeld University participated in the experiment in exchange for either course credit or 5€. Twenty-one participants were right handed (handedness score 0.97 ± 0.07), four left handed (handedness score −0.80 ± 0.08), and six ambidextrous (handedness score 0.02 ± 0.28) according to the revised Edinburgh inventory (Oldfield, 1971). Participants reported no known neuromuscular disorders and were naïve to the purpose of the study. Each participant read a detailed set of instructions on the task and provided written informed consent before the experiment. The study conformed to local ethics guidelines and the latest revision (World Medical Association, 2013) of the 1964 Declaration of Helsinki.

### Apparatus

**FIGURE 2:**
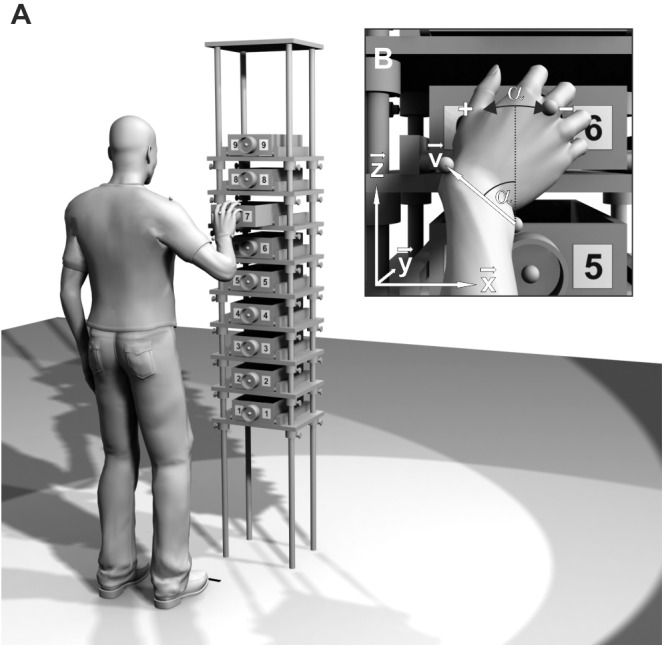
**(A)** Schematic of the experimental setup. Drawer height, spacing, and participant’s position are adjusted to the participant’s shoulder height and arm length. **(B)** Pro/supination angle α at the moment of drawer grasp. The projection of wrist vector **v** onto the drawer face (**x**-**z**-plane) is used to calculate α.

The apparatus used was a tall metal frame (222 cm high, 40 cm wide, and 30 cm deep) with nine wooden shelves (Figure 2A). A wooden drawer (8.5 cm high, 20 cm wide, and 30 cm deep; pullout range 21.5 cm) was placed on each shelf. At the center of each drawer front, a gray plastic ring with a diameter of 7 cm and a depth of 4 cm was affixed. To the left and right of the knob, a number from 1 (lowest) to 9 (highest) was attached.

### Preparation

Retroreflective markers were attached to four bony landmarks on the right arm of the participant: the most cranial point of the *acromion (AC)*, the *radial (RS)*, and *ulnar (US) styloid process*, and the back of the third *metacarpal (MC)*. The approximate height of the shoulder joint center (0.97 × height of *AC*) and length of the arm (between *AC* and *RS*) of the participant were measured in a t-pose (arms extended sideways and palms pointed forward).

The center of drawer #7 was aligned with the height of the shoulder joint center. Drawer spacing was set to a quarter arm length. The participant was positioned with the shoulder joint center one arm length in front of the setup and a third arm length to the left of the drawer centers. The participant’s position was marked by two strips of black tape: point of the toes and median plane of the body.

### Procedure

The experiment consisted of three tasks. Each task contained up to eight sequences of trials. A single trial was defined as the opening and closing of one drawer. Each trial started from an initial position, with the palm of the hand touching the thigh. The participant had to (1) raise the arm to the drawer, (2) grasp the handle with a five-finger grip, (3) fully open the drawer, (4) close the drawer, and (5) return to the initial position.

In Task 1, the participant performed four randomized sequences of the nine drawers (4 repetitions **×** 9 drawers; 36 trials). A list of pseudo-random (Mersenne twister algorithm; Matsumoto and Nishimura, 1998) permutations was created before the experiment. From the list, the experimenter announced the next drawer number as soon as the arm was back in the initial position.

In Task 2 and Task 3, the participants performed eight ordered sequences of trials, respectively: four ascending, four descending. Sequence order was alternated and counterbalanced across participants. The experimenter did not announce drawer numbers, but only the order of the sequence (“from top to bottom”/“from bottom to top”). The participant executed the trials of each sequence on their own.

In Task 2, a single sequence consisted of all nine drawers (2 sequences **×** 4 repetitions **×** 9 drawers; 72 trials). In Task 3, a single sequence consisted only of the drawers #1, #3, #5, #7, and #9. In both the ascending and descending sequences, participants had to skip every second drawer (2 sequences **×** 4 repetitions **×** 5 drawers; 40 trials).

Each participant conducted Task 1 first, to get accustomed to the experiment. Postures in randomized tasks are unaffected by motor hysteresis (Schütz et al., 2011) and, thus, should not affect behavior in the subsequent tasks. The order of Tasks 2 and 3 was counterbalanced across participants.

Before each task, the position of the participant in front of the apparatus was checked based on the floor marks. Participants had a resting period of 30 s between each sequence and of 2 min between each task. The entire experiment lasted approximately 45 min.

### Kinematic Analysis

Movement data were recorded by a Vicon MX (Vicon Motion Systems, Oxford, United Kingdom) motion capture system. Marker trajectories were reconstructed in Vicon Nexus 1.8.5, labeled manually, and exported to MATLAB (2008b, The MathWorks, Natick, MA, United States) for data analysis. The laboratory’s coordinate system was defined with the **x**-axis pointing to the right, the **y**-axis pointing to the front, and the **z-**axis pointing upward while standing in front of the apparatus (Figure 2B).

To identify the moment of drawer grasp for each trial, the **y**-component (perpendicular to the drawer face, see Figure 2B) of the *capitulum* marker (*MC*) was analyzed. Its trajectory started from a low initial value (the initial posture) and exhibited two local maxima before returning to the initial value. The first local maximum, which corresponded to the moment of drawer grasp, was used to calculate the pro/supination angle α.

For the calculation of α, the wrist axis was projected onto the drawer face (**x**-**z**-plane, see Figure 2B). A direction vector **v** was defined, pointing from *US* to *RS*: **v** = *RS* – *US*. From the vector components v_x_ and v_z_, the pro/supination angle α was calculated with the four-quadrant inverse tangent function of MATLAB. It was zero when the back of the hand pointed directly to the right (**v** pointed directly upward). Pronation of the hand caused an increase, supination a decrease of the pro/supination angle.

### Modeling

Previous studies’ results suggest that the pro/supination angle is a sigmoid function of drawer height (Schütz and Schack, 2013; Schütz et al., 2016, 2017). A more pronated posture is used for the descending and a more supinated posture for the ascending sequences. The hysteresis effect (difference between the a/descending sequences) is large at the central drawers and declines toward the periphery, where the ascending and descending grasp angle functions converge. This results in a significant main effect of “sequence” and an interaction of “sequence” × “drawer” (Schütz and Schack, 2013; Schütz et al., 2016).

A simple, five-parameter model was developed to capture the pattern of results found in a sequential drawer task.

**FIGURE 3:**
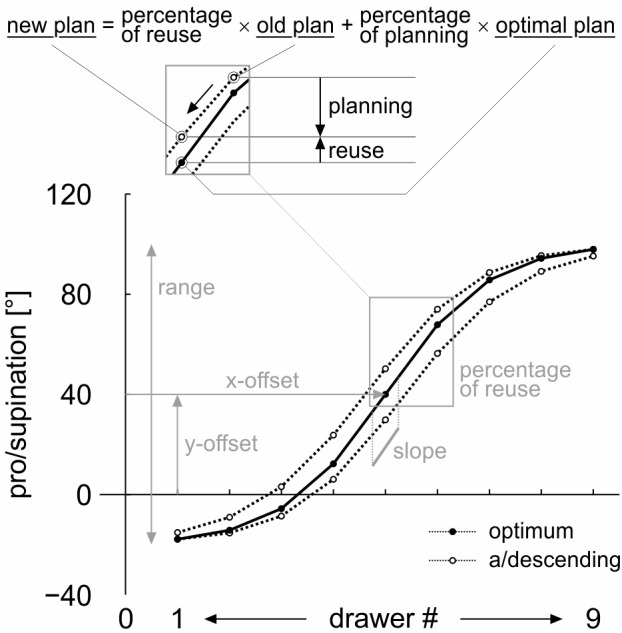
Parameters of the mathematical model. Four parameters describe the optimal pro/supination angle as a sigmoid function of drawer (height): its *range* (from lowest drawer to highest), its steepest *slope*, and the *x-* and *y-offset* of its origin. The sigmoid function is a hyperbolic tangent (*tanh*). The fifth parameter is a fixed *percentage of reuse* between subsequent drawers.

Four parameters are required to model the pro/supination angle as a function of the drawer number (Figure 3, *bottom*): the *range* of the sigmoid function (from lowest drawer to highest), its steepest *slope*, and the *x-* and *y-offset* of its origin. The sigmoid function itself is created by the hyperbolic tangent function (*tanh*) of MATLAB.

The fifth parameter is a fixed *percentage of (motor plan) reuse* between subsequent drawers.

The *cost-optimization model* (Schütz and Schack, 2013; Schütz et al., 2016) claims that, in an ordered task, each motor plan is created as a modification of the previous plan, with varying percentages of reuse and novel planning. The percentages depend on the cognitive cost of motor planning and the mechanical cost of motor execution. In a sequential drawer opening task, both cost factors and, thus, the percentages of motor plan reuse and novel planning, should be fairly constant across all drawers.

In our continuous posture selection task, the percentages of reuse and novel planning are reflected in the modification of the pro/supination angle between subsequent drawers. In Figure 3, *top*, the basic idea is demonstrated for the switch from drawer #6 to drawer #5 in the descending sequence. A *percentage of reuse* of 100 would result in the previous grasp posture (74.0°), used at drawer #6. A *percentage of reuse* of 0 (100% novel planning) would result in the optimal grasp angle (40.0°) for drawer #5. An intermediate *percentage of reuse* of 30 (Figure 3), for example, results in an intermediate grasp angle, calculated as 30% × 74.0° + 70% × 40.0° = 50.2°.

The model parameters were fitted to the measured data of individual participants using a least squares optimization algorithm (Levenberg, 1944; Marquardt, 1963). Before the fitting, the four repetitions for each condition were averaged to reduce variance. The goodness of fit was quantified both as the fraction of captured variance and as the root mean squared error (RMSE). Further, the standardized residuals were inspected visually to check for heteroscedasticity, outliers, and structural information not captured by the model.

In the randomized task, grasp angles were unaffected by motor hysteresis and, thus, should reflect the optimal grasp angle (Schütz et al., 2011). For our proposed model to be valid, the sigmoid fit created from four parameters (*percentage of reuse* = 0) should capture a major fraction of the total variance.

In an ordered task, the overall variance of the data is dominated by the main effect of “drawer.” Thus, for our proposed model to be valid for the ordered task, it should not only capture a major fraction of the overall variance, but also a major fraction of the hysteresis-specific variance, i.e., of the factor “sequence” and of the interaction of “sequence” × “drawer.” To this end, sums of squares are calculated for the model data and the measured data of each participant individually.

If a good fit is achieved, the fifth parameter returns the percentage of motor plan reuse in the ordered task. In theory, all model parameters could be modified arbitrarily by the optimization algorithm. The *percentage of reuse*, however, was limited to 0 as its lower bound.

Based on the percentage of reuse calculated from the ordered task, the size of the hysteresis effect in the skipped ordered task is predicted. In the skipped ordered task, the distance between subsequent drawers is larger than in the ordered task. Therefore, the size of the hysteresis effect should be larger. The hysteresis effect size in the skipped ordered task is compared to the effect size at the equivalent drawers (#1, #3, #5, #7, and #9) in the ordered task.

To estimate the impact of “drawer” on the total variance in the ordered task and to compare the size of the hysteresis effect in the ordered and the skipped ordered task, repeated measures analyses of variance (rmANOVAs) were calculated on the pro/supination angles. The four repetitions for each condition were averaged to reduce variance. The rmANOVAs were calculated in SPSS (22, IBM Corp., Armonk, NY, United States) to apply the Greenhouse–Geisser correction to the *p*-values where appropriate.

## Results

**FIGURE 4:**
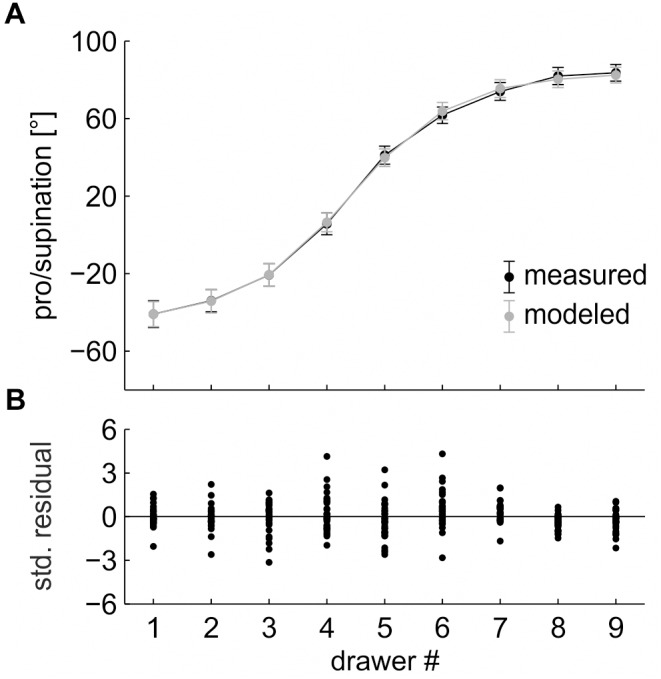
**(A)** Pro/supination angle of the measured data (*black line*) and the model fit (*gray line*). *Each data point* represents the average across the factors “repetition” and “participant,” split by “drawer.” *Error bars* indicate 95% (within-subject) confidence intervals. **(B)** Standardized residuals. *Each data point* represents the average across the factor “repetition,” split by “drawer” and “participant.”

The four-parameter model was applied to the randomized data of Task 1. The model captured 99.3 ± 0.6% (SD) of the total variance and reached an RMSE of 3.7 ± 2.1° (Figure 4A). The standardized residuals showed no heteroscedasticity (increasing variance with increasing *x*-value) and no marked outliers (Figure 4B). They were normally distributed and had no non-linear structure, which would indicate that a different (e.g., linear, quadratic) model could provide a better fit (Ellis and Duggleby, 1978; Straume and Johnson, 1992). Overall, the sigmoid model provides a valid fit of the randomized data.

For the four model parameters, the fitting algorithm returned the following values: *range* = 133.3 ± 32.8°, *slope* = 39.4 ± 18.9°/drawer, *y-offset* = 17.2 ± 15.5°, and *x-offset* = 4.4 ± 0.8 (drawers).

**FIGURE 5:**
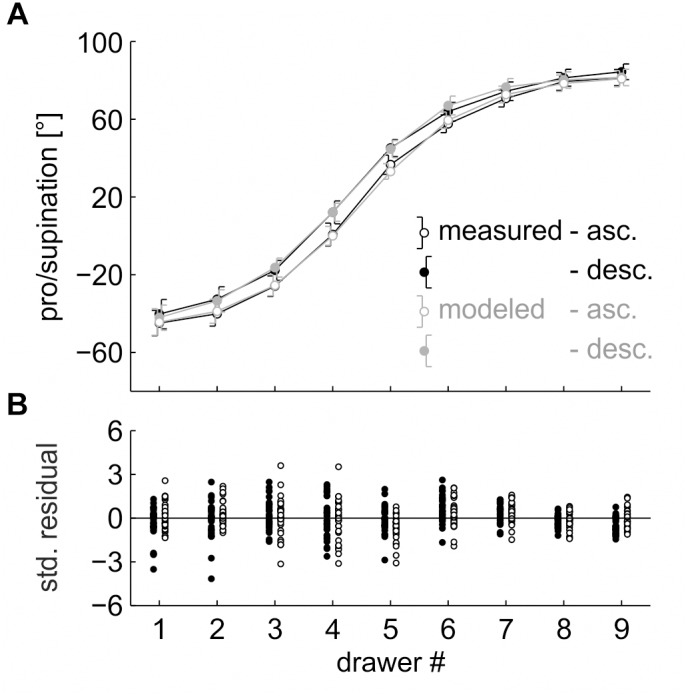
**(A)** Pro/supination angle of the measured data (*black lines*) and the model fit (*gray lines*). *Each data point* represents the average across the factors “repetition” and “participant,” split by “sequence” and “drawer.” *Error bars* indicate 95% (within-subject) confidence intervals. **(B)** Standardized residuals. *Each data point* represents the average across the factor “repetition,” split by “sequence,” “drawer,” and “participant.”

After confirming the validity of the sigmoid fit with the randomized data, the five-parameter model was applied to the ordered data of Task 2. The model captured 98.6 ± 1.6% of the total variance and reached an RMSE of 4.8 ± 1.7° (Figure 5A). Again, the standardized residuals showed no heteroscedasticity were normally distributed and had no non-linear structure (Figure 5B).

To test whether the total variance was dominated by the factor “drawer,” a 2 (sequence) × 9 (drawer) rmANOVA was calculated on the measured pro/supination angles, with “sequence” and “drawer” as within-subject factors. The main effect of “sequence,” *F*(1,30) = 31.603, *p* < 0.001, η^2^ = 0.003, the main effect of “drawer,” *F*(8,240) = 407.745, *p* < 0.001, η^2^ = 0.857, and the interaction of “sequence” × “drawer,” *F*(8,240) = 2.860, *p* = 0.036, η^2^ = 0.001, were significant (Figure 5A). Total variance was dominated by “drawer.”

To evaluate the model fit with respect to the motor hysteresis effect only, we calculated the combined fraction of variance of “sequence” and “sequence” × “drawer.” The model captured only 46.8 ± 38.2% of this variance. A closer inspection of individual fits revealed that a number of participants exhibited no effect of “sequence” and that the interaction of “sequence” × “drawer” in reality reflected random noise on the grasp angles instead of a modulation of the hysteresis effect across drawers.

We therefore restricted the analysis to participants with an actual hysteresis effect. To this end, we calculated the ratio between the variance of “sequence” and the variance of “sequence” × “drawer.” The top 15 participants were classified as “hysteresis,” the bottom 15 participants were classified as “noise.” In the “noise” group, only 16.0 ± 28.8% of the combined variance was captured. In the “hysteresis” group, the model captured 76.0 ± 18.0% of the combined variance. If a hysteresis effect was present, the proposed model achieved a decent fit.

The model parameters were similar to those of the randomized task: *range* = 131.9 ± 29.9°, *slope* = 41.2 ± 20.3°/drawer, *y-offset* = 16.5 ± 15.2°, and *x-offset* = 4.4 ± 0.6 (drawers). Paired, two-tailed *t*-tests showed that none of the parameters differed from their randomized counterparts (all |*t*(30)|≤ 0.827, all *p* ≥ 0.415). The fifth parameter, *percentage of reuse*, was 15.6 ± 13.0. Only 15.6% of the previous motor plan was reused on average, whereas 84.4% of the plan was modified. The large standard deviation reflects the inter-individual differences in hysteresis effect described previously.

**FIGURE 6:**
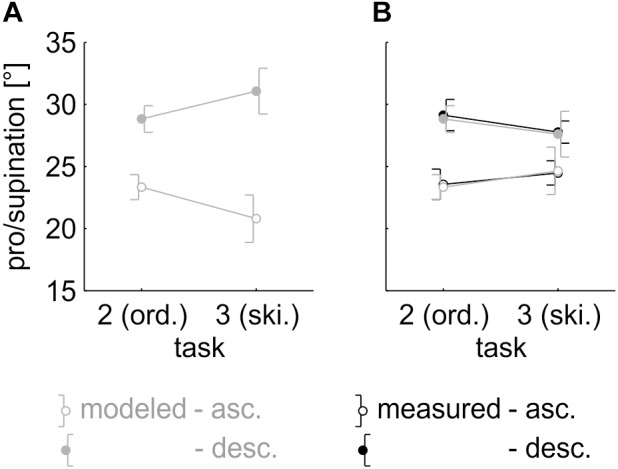
**(A)** Model data for the ordered task and model prediction for the skipped ordered task (if percentage of reuse was constant across tasks). *Each data point* represents the average across the factors “drawer” (drawers #1, #3, #5, #7, and #9 only), “repetition,” and “participant,” split by “sequence.” *Error bars* indicate 95% (within-subject) confidence intervals. **(B)** Pro/supination angle of the measured data (*black lines*) and the model fit (*gray lines*). *Each data point* represents the average across the factors “drawer” (drawers #1, #3, #5, #7, and #9 only), “repetition,” and “participant,” split by “sequence.” *Error bars* indicate 95% (within-subject) confidence intervals.

Using the model parameters from the ordered task, we predicted the size of the hysteresis effect in the skipped ordered task (Task 3). Due to the larger differences between the previous and the optimal posture, we expected a larger hysteresis effect in the skipped ordered task. The model predicted an average difference of 10.3° between the ascending and descending sequences in the skipped ordered task, as opposed to an average difference of 5.6° at drawers #1, #3, #5, #7, and #9 in the ordered task (Figure 6A).

A 2 (task) × 2 (sequence) rmANOVA was calculated on the pro/supination angles measured in both tasks to verify this prediction, with “task” and “sequence” as within-subject variables. The grasp angles at drawers #1, #3, #5, #7, and #9 were averaged. We expected a significant interaction of “task” × “sequence” and a larger hysteresis effect in the skipped ordered task. The main effect of “sequence,” *F*(1,30) = 50.682, *p* < 0.001, η^2^ = 0.025, and the interaction of “task” × “sequence,” *F*(1,30) = 5.436, *p* = 0.027, η^2^ = 0.002 were significant. The size of the hysteresis effect differed depending on the task. Effect direction, however, was in contrast to the model prediction: in the skipped ordered task, the size of the hysteresis effect decreased to 3.3° (Figure 6B).

The five-parameter model was applied to the skipped ordered data. The model captured 99.2 ± 1.0% of the total variance and reached an RMSE of 3.6 ± 1.5° (Figure 6B). Model parameters were: *range* = 131.4 ± 33.1°, *slope* = 37.3 ± 14.0°/drawer, *y-offset* = 16.6 ± 16.6°, *x-offset* = 4.3 ± 0.7, and *percentage of reuse* = 6.1 ± 6.8. Paired, two-tailed *t*-tests showed that the first four parameters did not differ from the ordered task (all |*t*(30)|≤ 1.225, all *p* ≥ 0.230). The general grasping behavior was the same in both tasks. For the fifth parameter, *percentage of reuse*, there was a significant difference, *t*(30) = 4.646, *p* < 0.001. Participants reused a significantly smaller percentage of the previous motor plan in the skipped ordered task.

## Discussion

In the current study, we asked whether, in a sequential drawer opening task, (1) the optimal grasp angle was a sigmoid function of drawer (height), (2) the percentage of motor plan reuse was constant across all drawers, and whether (3) such a constant percentage of reuse across all drawers resulted in a larger hysteresis effect at the central and a smaller hysteresis effect at the peripheral drawers. To this end, participants had to execute three sequential drawer opening tasks: a randomized task, an ordered (a/descending) task where every drawer was opened, and an ordered task where every second drawer was opened. The pro/supination angle of the hand was measured as the dependent variable.

A simple model was created to capture the variance of the pro/supination angle in all three tasks. The model assumes that optimal grasp angle is a sigmoid function of drawer (height), as has been implied in previous drawer studies (Weigelt et al., 2009; Schütz et al., 2011, 2016, 2017; Schütz and Schack, 2013). A sigmoid posture function was also found in studies where targets had to be contacted in a vertical progression (Rosenbaum and Jorgensen, 1992; Short and Cauraugh, 1997, 1999). In the current study, the optimal grasp angle was measured in the randomized task (Task 1). In randomized sequences of trials, postures are unaffected by the previous posture (Schütz et al., 2011). Therefore, participants should create each motor plan from scratch and, thus, adopt the (mechanically) optimal posture at each drawer.

For our first assumption to hold true, the reduced (four-parameter, no *percentage of reuse*) version of the model should capture a major fraction of the total variance in the randomized task (Task 1). Indeed, the model captured 99.3 ± 0.6% (SD) of the total variance. More importantly, the standardized residuals had no non-linear structure, which would indicate that a different (e.g., linear) model could provide a better fit of the data. Both findings support the notion that the optimal grasp angle in a sequential drawer opening task follows a sigmoid function.

As the sigmoid function is a good estimate of the optimal grasp angle, the parameters returned by the fitting algorithm can be used to interpret the grasping behavior. A *range* of 133.3 ± 32.8° is within the viable pro/supination range of the forearm (cf. Boone and Azen, 1979) and, thus, anatomically plausible. The *slope* of 39.4 ± 18.9°/drawer indicates the maximum change in posture between two adjacent drawers. This maximal change is located at an *x-offset* of 4.4 ± 0.8 drawers, i.e., between drawers #4 and #5 (cf. Figure 4A). An *x-offset* below 5.0 (the central drawer) indicates that the sigmoid function is not point-symmetrical and, thus, has a steeper slope at the bottom compared to the top drawer. If our second assumption, a fixed percentage of reuse across drawers, was valid, we would expect a larger hysteresis effect at the bottom drawer in the ordered task.

The pro/supination angles in the ordered task (Task 2) follow the pattern of results found in previous drawer studies (Weigelt et al., 2009; Schütz et al., 2011, 2016; Schütz and Schack, 2013), with a main effect of “sequence” (hysteresis) and an interaction of “sequence” × “drawer” (larger hysteresis effect at the central drawers). As suggested by the *x-offset* parameter of Task 1, the size of the hysteresis effect was slightly larger at the bottom drawers than at the top drawers. For our second and third assumption to hold true, a full (five-parameter, fixed *percentage of reuse*) version of the model should capture a major fraction of the total variance in Task 2. Indeed, the model captured 98.6 ± 1.6% of the variance.

As the total variance was dominated by the main effect of “drawer,” the model should also explain a major fraction of the combined variance of the factor “sequence” and of the interaction “sequence” × “drawer,” the two variance components affected by hysteresis. Of this combined variance, the model only captured 46.8 ± 38.2%. A closer inspection of individual participants showed that a number of participants had no main effect of “sequence,” i.e., they exhibited no hysteresis effect. The tendency to reuse the previous motor plan seems to differ strongly across participants, as is reflected by the high standard deviation [15.6 ± 13.0 (SD)] found for the *percentage of reuse* parameter.

In participants with low/no hysteresis effect, the interaction of “sequence” × “drawer” did not reflect a modulation of hysteresis effect size across drawers, but instead reflected random noise, which could not be captured by the model. If the analysis was restricted to participants *with* a hysteresis effect, the model captured 76.0 ± 18.0% of the combined variance and, thus, achieved a decent fit of the data. If a hysteresis effect is present, a model that assumes a constant *percentage of reuse* across all drawers can reproduce the hysteresis pattern reported in previous studies (larger hysteresis effect at the central drawers, declining effects toward the periphery; Weigelt et al., 2009; Schütz et al., 2011, 2016; Schütz and Schack, 2013).

None of the four parameters for the sigmoid (optimal grasp) function differed from their randomized counterparts, which supports the notion that optimal grasp angles are stable across experiments and reflect behavioral data and not just abstract fitting parameters. The additional fifth parameter, the *percentage of reuse*, was 15.6 ± 13.0, which implies that only a small fraction of the previous motor plan was actually reused, whereas 84.4% of the plan was modified. According to the *cost-optimization model* (Schütz et al., 2016), a low percentage of reuse indicates that the cognitive cost of motor planning is small compared to the mechanical cost of motor execution.

This finding has important implications for the future study of cost-optimization. Schütz and Schack (2013) found significant effects of a change in mechanical cost on the hysteresis effect. If mechanical cost of motor execution is such a predominant cost factor, however, it will be more difficult or even impossible to corroborate a comparable effect by a change in cognitive cost.

If the percentage of reuse was constant and depended only on the cognitive and mechanical cost of the movement, the size of the hysteresis effect should increase with the difference in optimal posture between subsequent drawers. We therefore expected a larger hysteresis effect in the skipped ordered task (Task 3). Results showed a significant interaction of “task” × “sequence” between Tasks 2 and 3, indicating that the size of the hysteresis effect differed depending on the task. However, in contrast to our prediction, the hysteresis effect actually decreased in the skipped ordered task. While this result is in contrast to the prediction, it does not invalidate the model, but instead shows that the assumption of a constant percentage of reuse across different tasks was flawed.

When applied to the skipped ordered data of Task 3, the five-parameter model captured 99.2 ± 1.0% of the total variance and, thus, was able to replicate the measured pattern of results almost perfectly. The first four parameters were similar to the ordered task (Task 2), lending further support to the idea that a sigmoid optimality function is a stable characteristic of vertical reaching. The fifth parameter, the *percentage of reuse*, was significantly smaller (6.1 ± 6.8) than in Task 2. Participants reused a smaller percentage of the previous motor plan in the skipped ordered task than in the ordered task, which shows that reuse is not constant across tasks. Percentage of reuse seems to not only depend on the cognitive and mechanical cost of the movement, but on other factors not yet taken into account by the model, like the delay between trials.

Jax and Rosenbaum (2007, 2009) found that hysteresis effects on the hand path decayed quickly as the time between trials increased. In the current study, however, participants did not move their hand directly from one drawer to the next, but returned to an initial position between trials. Therefore, time between trials was the same in the ordered and the skipped ordered task. By asking participants to skip every second drawer, however, a number of other factors were modified, such as (1) the spatial difference between subsequent drawers, (2) the sequence of digits (#1, #3, #5, etc.), and (3) the number of drawers in a sequence. All of these factors could result in a reduction of the perceived closeness of subsequent drawers and an increase in novel planning. To determine which context factors affect the percentage of reuse, and to what extent, will require a series of systematic experiments in the future.

In conclusion, we showed that a simple model with a sigmoid optimal grasp angle function and a fixed percentage of reuse could capture a major fraction of the variance in a randomized and an ordered sequential drawer opening task. This finding supports the notion that (1) the optimal pro/supination angle is a sigmoid function of drawer (height), (2) the percentage of motor plan reuse is constant across drawers, and (3) a constant percentage of reuse results in a larger hysteresis effect at the central drawers. A smaller percentage of reuse was found in the skipped ordered task. This indicates that motor planning is not only affected by the cognitive and mechanical cost of a movement, but by other factors (e.g., task context) as well.

While the proposed model in the current study was applied to single, well-established hysteresis task, it should be applicable to arbitrary sequential tasks and provide a sensitive tool to measure changes in the percentage of reuse under different experimental manipulations. Especially in sequential posture selection tasks, where hysteresis effects blend with an optimal posture that differs from trial to trial, the current model offers a straightforward approach to isolate the hysteresis effect and to estimate the amount of novel motor planning.

## Data Availability

The datasets generated for this study are available on request to the corresponding author.

## Ethics Statement

This study was carried out in accordance with the recommendations of the Bielefeld University ethics committee and the guidelines of the DGPs. All subjects gave written informed consent before the experiment in accordance with the Declaration of Helsinki. The protocol was approved by the Bielefeld University ethics committee.

## Author Contributions

CS and TS conceived the idea and contributed to the final version of the manuscript. CS planned and carried out the experiments and the simulations, and conducted the statistical analyses.

## Conflict of Interest Statement

The authors declare that the research was conducted in the absence of any commercial or financial relationships that could be construed as a potential conflict of interest.
